# Prevalence and prognosis of synchronous distant metastatic tonsil squamous cell carcinomas

**DOI:** 10.7150/ijms.50966

**Published:** 2021-01-01

**Authors:** Yujiao Li, Chaosu Hu

**Affiliations:** 1 Department of Radiation Oncology, Fudan University Shanghai Cancer Center, Shanghai, China.; 2 Department of Oncology, Shanghai Medical College, Shanghai, China.

**Keywords:** synchronous distant metastases, tonsil squamous cell carcinomas, metastatic pattern, prevalence, prognosis

## Abstract

Background: To analyze the prevalence proportions and prognostic factors of synchronous distant metastases in patients with tonsil squamous cell carcinomas (TSCC).

Methods: TSCC patients were extracted from the Surveillance, Epidemiology and End Results (SEER) database between 2010 and 2014. We examined the association between clinical manifestations and distant metastases using Chi-squared tests. Predictors of 5-year survival were assessed using univariate and multivariate analyses.

Results: A total of 6193 patients were analyzed and lung was the most common site of distant metastases. Poorly/undifferentiated differentiation was found to be significantly correlated with lung metastasis (p=0.033) and liver and bone metastases were associated with African American (p=0.000 and p=0.000, respectively). A higher T classification was associated with higher prevalence of lung, liver, bone and brain metastasis (p=0.000, p=0.000, p=0.000 and p=0.007, respectively). The same results were found in N classification in lung, liver, and bone metastasis (p=0.000, p=0.000, and p=0.000, respectively). Worse prognosis was associated with older age, Blacks, lower grade, higher T and N classification, no surgery therapy and more metastatic sites.

Conclusion: Lung was the most frequent lesion of synchronous distant metastases and liver and bone metastases were associated with African American. Higher T and N classification were independent prognostic parameters for higher prevalence of lung, bone, liver and brain metastasis. Worse prognosis was associated with older age, African Americans, lower grade, higher T and N classification, no surgery therapy and more metastatic sites.

## Introduction

Tonsil squamous cell carcinoma (TSCC) is one of the most common oropharyngeal neoplasm and the incidence rates of TSCC have significantly increased in recent decades 1-3. Several studies have described the association between the clinicopathological characteristics, including age, tumor size, tumor grade, tumor stages at presentation, and lymph node or distant metastases and clinical outcomes of TSCC. Up-front surgery followed by adjuvant therapy, if appropriate, or radiotherapy alone has been the principal treatment modality for early-stage TSCC and postoperative radiotherapy has been used for close or positive resection margins, T3-4 tumors, and neck node metastases 4.

Metastases are the main cause of human cancer deaths, and its treatment continues to be a major challenge. The identification of factors associated with distant metastases is of paramount relevance for therapeutic planning. Our goals were to analyze the prevalence proportions and prognosis of synchronous distant metastases in patients with TSCC using the Surveillance, Epidemiology, and End Results (SEER) database.

## Materials and Methods

### Cohort population

We obtained data from the current SEER database, which consists of 18 population-based cancer registries. This database collects and publishes cancer prevalence and survival data covering approximately 28% of the total population in the United States. SEER*Stat Version 8.3.4 (http://www. seer.cancer.gov/seerstat) from the National Cancer Institute was used to identify eligible patients in this study. Because the SEER database began collecting information on the presence or absence of metastases at the time of diagnosis in 2010, we included patients diagnosed with microscopically confirmed TSCC between 1 January 2010 and 31 December 2014. We selected patients with only one primary malignancy in their lifetime. We excluded patients mainly because of lack of pathology type of tumor, unknown racial information, unstaged tumors or 'blanks' metastatic site. In total, 6193 TSCC patients were eligible for inclusion in the prevalence analyses.

### Statistical analysis

Descriptive statistics were used to examine the baseline characteristics of the patients. The primary study outcomes were overall survival (OS) and cancer-specific survival (CSS). OS was defined as time to the date of death due to any cause or the date of last follow-up. CSS was defined as time from initial treatment to death due to cancer. Kaplan-Meier survival curves were compared using the log-rank test. Hazard analysis was conducted using the Cox proportional hazards model. SPSS software, version 22.0 (SPSS, Chicago, IL, USA) was used for additional data processing. A probability value (*p* value) of *<* 0.05 was considered statistically significant for all tests.

## Results

### Clinical Characteristics of all patients

**Figure 1:**
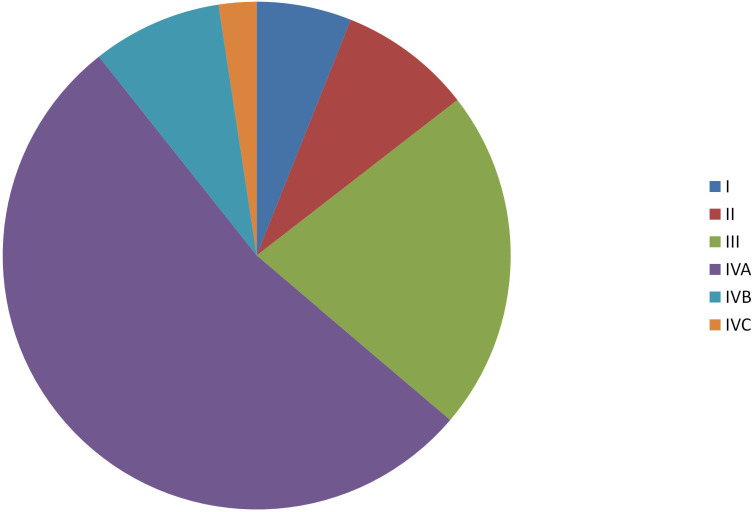
Distribution of tumor stages.

Data for a total of 6193 patients, including 5083 males and 1110 females, were investigated. The median age was 59 months (range, 17-99 months). Among the cohort of the patients, 4.4%, 40.6% and 55.0% tumors were well differentiated (Grade I), moderately differentiated (Grade II) and poorly/undifferentiated differentiated (Grade III), respectively. About 63.8% of the patients were stage IV (**Figure 1**).

According to the 7^th^ edition of UICC/AJCC Staging System, 1835 patients (29.6%) were T1, 2484 patients (40.1%) were T2, 1023 patients (16.5%) were T3, 566 patients (9.1%) were T4a, and 285 patients (4.6%) were T4b. With regard to N classifications, 1259 patients (20.3%) were N0, 1296 patients (20.9%) were N1, 719 patients (11.6%) were N2a, 2087 patients (33.7%) were N2b, 555 patients (9.0%) were N2c, and 277 patients (4.5%) were N3. Patients' characteristics are listed in **Table 1.**

### Metastasis pattern

At the time of diagnosis, there were 17 patients with liver metastasis, 67 patients with lung metastasis, 37 patients with bone metastasis and 6 patients with brain metastasis.

As shown in **Table 2**, tumor grade was found to be significantly correlated with lung metastasis and poorly/undifferentiated differentiation was an independent prognostic parameter for higher prevalence of lung metastasis (*p*=0.033). What's more, we observed that T classification was also an independent parameter for metastatic diseases. A higher T classification was associated with higher prevalence of lung, bone, liver and brain metastasis (*p*=0.000, *p*=0.000, *p*=0.000 and *p*=0.007, respectively). The same results were found in N classification in lung, bone, and liver metastasis (*p*=0.000, *p*=0.000, and *p*=0.000, respectively). In addition, liver and bone metastases were associated with African American (*p*=0.000 and *p*=0.000, respectively).

### Survival

The overall mean follow-up of all patients in the cohort was 21.0 months (range, 0-59 months). In the univariate analysis, age, gender, race, grade, primary site, T category, N category, distant metastases and surgery therapy were significantly associated with OS and CSS (*P* < 0.05).

Gender, age, race, grade, primary site, T classification, N classification, history of surgery and distant metastases were selected in the multivariate model. Age, race, grade, primary site, T classification, N classification, surgery therapy and distant metastases were all independent prognostic factors in the multivariable analysis for CSS (**Table 3**). Elderly black patients with higher T or N classification, multiple sites of metastases and no surgical therapy to primary tumor were more likely to reduce life expectancy.

**Figure 2:**
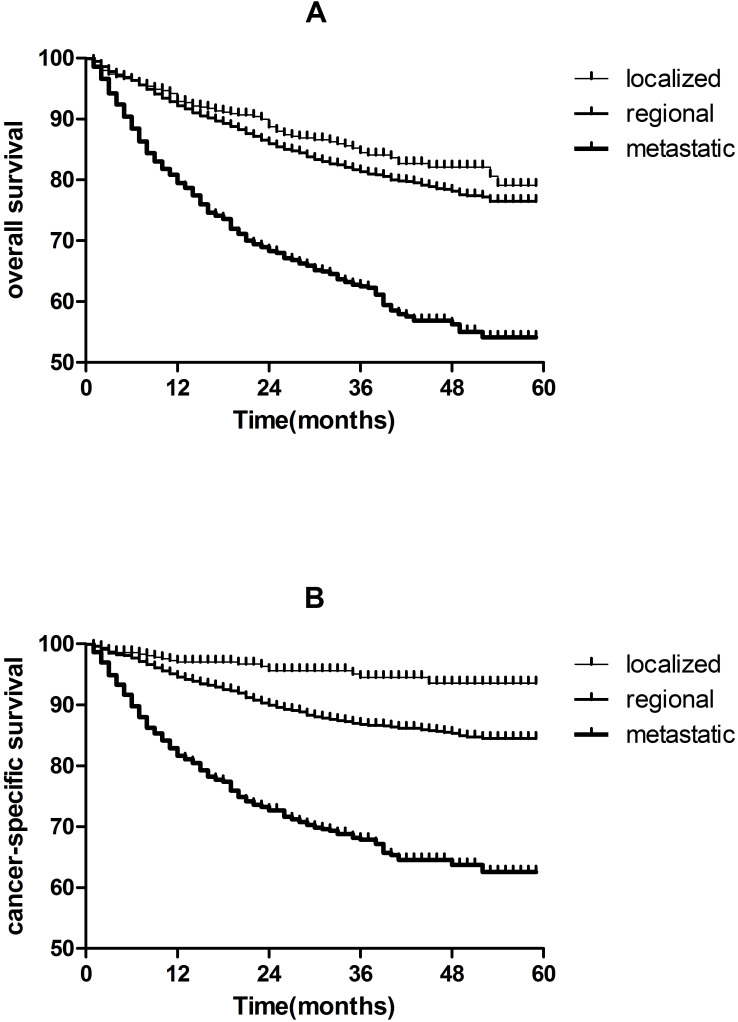
Kaplan-Meier analysis of overall survival and cancer-specific survival in localized, regional and metastatic tonsil squamous cell carcinomas patients. A, overall survival in localized, regional and metastatic tonsil squamous cell carcinomas patients (P < 0.05). B, cancer-specific survival in localized, regional and metastatic tonsil squamous cell carcinomas patients (P < 0.05).

As shown in **Figure 2**, 5-year CSS are 93.6% and 84.5% for localized and regional TSCC patients, respectively, which are much higher than metastatic TSCC patients (62.6%). The same results were found in OS.

## Discussion

In this study, 6193 patients with TSCC were evaluated and 86 (0.13%) patients had synchronous distant metastases at diagnosis. In the present series, the niches of distant metastasis were predominantly located in the lungs, bones, liver and brain, in accordance with the literature 5. We observed some interesting relationships between the clinicopathological characteristics and distant metastases patterns. For instance, liver and bone metastases were associated with African American. Nabil et al. found that TSCC in white men is likely human papillomavirus (HPV) driven, and Frederic et al. found that HPV-negativity are factors associated with the development of distant metastases, which could partly explain the observed ethnic-based differences 6, 7. However, absence of information about HPV and/or p16 status of the patients in the current analysis prevented us to adjust our analyses for these important factors. What's more, advanced T classifications increased the risk of distant metastases and advanced regional disease (N2 or N3 disease) are found in patients with M1 disease, which were consistent with many other studies 7-10.

A number of variables influencing the prognosis of TSCC have been described previously (**Table 3**). In the present study, eight clinicopathological factors were found to be associated with patient OS by univariate analysis, including age, race, grade, primary site, T classification, N classification, distant metastases and surgery therapies. Based on the present study, Caucasian was associated with a better prognosis than Asian and African American. In terms of age, several reports have shown that patients' age is one of the prognostic factors in oropharyngeal carcinoma including TSCC 11, 12. The differential protective effect of marriage based on HPV infection, which correlated with favorable prognosis, was more frequently observed in younger Caucasian patients than in the elderly 13; therefore, old aged African American could be secondary surrogates of poor tumor biology which is unrelated to HPV infection. Unfortunately, because the HPV and/or p16 status of patients in the present study was unknown, this hypothesis could not be tested. Regarding that many recent studies for altering therapy based on HPV status are in progress, the lack of details of HPV status in this study has significant limitations 14.

Furthermore, early diagnosis and treatment are particularly important; especially the incidence of TSCC has increased in past 3 decades 2, 3, 15, 16. We observed that 5-year CSS are 93.6% and 84.5% for localized and regional TSCC patients, respectively, which are much higher than metastatic TSCC patients(62.6%). we observed the same pattern of associations for OS (**Figure 2**).

This is the first population-based analysis assessing prevalence proportion and prognostic factors of distant metastases of TSCC patients. Some limitations of our study should be acknowledged. First of all, the SEER database only provides information about four sites of metastases at diagnosis: bone, brain, lung, and liver. We don't have information of metastasis to other sites, which may influence the prognostic assessment of the metastases group. Second, the lack of data on additional predictors of OS such as HPV and/or p16 status, performance status, comorbidities, chemotherapy and/or postoperative radiotherapy, positive surgical margins at final pathology prevented us to adjust our analyses for these important factors.

## Conclusion

Lung was the most frequent lesion of synchronous distant metastases and liver and bone metastases were associated with African American. Higher T and N classification were independent prognostic parameters for higher prevalence of lung, bone, liver and brain metastasis. Worse prognosis was associated with older age, African Americans, lower grade, higher T and N classification, no surgery therapy and more metastatic sites.

## Figures and Tables

**Table 1:** Clinical manifestations of 6193 patients

	Number of patients	%
**Age**		
Median	59	
Range	17-99	
**Gender**		
Male	5083	82.1
Female	1110	17.9
**Race**		
Caucasian	5447	88.0
Asian	221	3.6
African American	525	8.5
**T classification**		
T1	1835	29.6
T2	2484	40.1
T3	1023	16.5
T4a	566	9.1
T4b	285	4.6
**N classification**		
N0	1259	20.3
N1	1296	20.9
N2a	719	11.6
N2b	2087	33.7
N2c	555	9.0
N3	277	4.5
**Grade**		
1	272	4.4
2	2512	40.6
3	3409	55.0
**Surgery therapy**		
Yes	3401	54.9
No	2792	45.1
**Tumor location**		
Tonsillar fossa	770	12.4
Tonsillar pillar	380	6.1
Overlapping lesion of tonsil	61	1.0
Tonsil, NOS	4982	80.4
**Metastasis patterns**		
No metastasis site	6091	98.4
One metastasis site	83	1.3
Multiple metastasis sites	3	0.3

Abbreviations: NOS: not otherwise specified.

**Table 2:** Demographic characteristics of patients with and without metastases

Features	Lung Metastasis		*P* value	Liver Metastasis		*P* value	Bone Metastasis		*P* value	Brain Metastasis		*P* value
	No	Yes		No	Yes		No	Yes		No	Yes	
**Gender**			0.076			0.021			0.081			0.294
Male	5034	49		5073	10		5057	26		5079	4	
Female	1092	18		1103	7		1099	11		1108	2	
**Age**			0.462			0.149			0.248			0.426
≤59	3274	39		3307	6		3297	16		3311	2	
>59	2852	28		2869	11		2859	21		2876	4	
**Race**			0.143			0.000			0.000			0.086
Caucasian	5393	54		5436	11		5424	23		5443	4	
Asian	218	3		221	0		219	2		221	0	
African American	515	10		519	6		513	12		523	2	
**Grade**			0.033			0.184			0.360			0.783
1	272	0		272	0		272	0		272	0	
2	2476	36		2508	4		2498	14		2510	2	
3	3378	31		3396	13		3386	23		3405	4	
**T classification**			0.000			0.000			0.000			0.007
T1	1826	9		1833	2		1829	6		1835	0	
T2	2470	14		2480	4		2470	14		2481	3	
T3	1006	17		1021	2		1019	4		1023	0	
T4a	548	18		558	8		557	9		563	3	
T4b	276	9		284	1		281	4		285	0	
**N classification**			0.000			0.000			0.000			0.527
N0	1256	3		1258	1		1253	6		1257	2	
N1	1288	8		1293	3		1292	4		1295	1	
N2a	716	3		719	0		718	1		719	0	
N2b	2069	18		2083	4		2078	9		2086	1	
N2c	529	26		549	6		542	13		554	1	
N3	268	9		274	3		273	4		276	1	
**Tumor location**			0.937			0.130			0.033			0.712
Tonsillar fossa	763	7		769	1		767	3		769	1	
Tonsillar pillar	376	4		378	2		379	1		379	1	
Overlapping lesion of tonsil	60	1		60	1		59	2		61	0	
Tonsil, NOS	4927	55		4969	13		4951	31		4978	4	

Abbreviations: NOS: not otherwise specified.

**Table 3:** Multivariable logistic regression for OS and CSS of TSCC in the SEER cohort

Prognostic factor	Overall survival				Cancer-specific survival			
	*P* value	HR	Lower CI	Higher CI	*P* value	HR	Lower CI	Higher CI
Age	0.000	1.843	1.625	2.090	0.000	1.738	1.477	2.046
**Gender**	0.344				0.251			
Male		1 (reference)				1 (reference)		
Female		1.054	0.904	1.229		1.094	0.893	1.341
**Race**	0.000				0.000			
Caucasian		1 (reference)				1 (reference)		
Asian		1.231	0.894	1.694		1.256	0.847	1.860
African American		1.694	1.432	2.003		1.663	1.328	2.082
**Grade**	0.000				0.000			
1		1 (reference)				1 (reference)		
2		0.789	0.610	1.020		0.649	0.454	0.927
3		0.575	0.443	0.746		0.439	0.306	0.629
**T classification**	0.000				0.000			
T1		1 (reference)				1 (reference)		
T2		1.172	0.971	1.415		1.192	0.914	1.555
T3		1.753	1.422	2.161		1.860	1.397	2.476
T4a		2.591	2.084	3.220		3.032	2.257	4.072
T4b		3.181	2.471	4.094		3.563	2.557	4.964
**N classification**	0.285				0.000			
N0		1 (reference)				1 (reference)		
N1		0.843	0.696	1.021		1.228	0.926	1.628
N2a		0.684	0.519	0.901		0.838	0.562	1.248
N2b		0.828	0.697	0.984		1.167	0.903	1.508
N2c		1.121	0.907	1.386		1.613	1.208	2.154
N3		1.229	0.935	1.615		2.004	1.421	2.826
**Tumor location**	0.000				0.003			
Tonsillar fossa		1 (reference)				1 (reference)		
Tonsillar pillar		1.145	0.889	1.474		1.100	0.784	1.544
Overlapping lesion of tonsil		0.964	0.568	1.636		0.823	0.414	1.636
Tonsil, NOS		0.819	0.695	0.965		0.790	0.635	0.982
Surgery therapy	0.000	1.890	1.636	2.183	0.000	2.276	1.859	2.788
**Distant metastases**	0.000				0.000			
No		1 (reference)				1 (reference)		
Single		3.790	2.821	5.093		4.972	3.586	6.893
Multiple		8.192	4.844	13.854		12.145	6.799	21.695

Abbreviations: CI, confidence interval; HR, hazard ratio; *P* values were calculated using an adjusted Cox proportional-hazards model; NOS, not otherwise specified.
